# Continuous Monitoring of Intra-abdominal Pressure in Severe Acute Pancreatitis Leads to Early Detection of Abdominal Compartment Syndrome: A Case Report

**DOI:** 10.7759/cureus.24606

**Published:** 2022-04-29

**Authors:** Olutola Akiode, Vanessa Moll, Gregory Schears

**Affiliations:** 1 Department of Internal Medicine, Wyoming Medical Center, Casper, USA; 2 Anesthesiology, Emory University School of Medicine, Atlanta, USA; 3 Department of Anesthesiology and Perioperative Medicine, Mayo Clinic, Rochester, USA

**Keywords:** intra-abdominal pressure (iap), acute kidney injury (aki), abdominal compartment syndrome (acs), acute pancreatitis, urinary output monitoring, iap monitoring, continuous monitoring, acute kidney injury

## Abstract

Acute pancreatitis is a risk factor for intra-abdominal hypertension (IAH) and abdominal compartment syndrome (ACS). Immediate detection and management of IAH and ACS are critical for patient survival. Obtaining accurate and consistent intra-abdominal pressure and urinary output with high frequency is challenging, but critical for effective patient management. The presented case is of a 40-year-old man with a history of chronic alcoholism who developed severe acute pancreatitis. The patient was fluid resuscitated for distributive shock; hypoxic respiratory failure, intubation, and anuria followed. Real-time monitoring of urinary output and intra-abdominal pressure (IAP) allowed for early recognition of acute kidney injury (AKI) and ACS leading to early surgical intervention. Normalized IAP returned renal function and re-establishment of stable hemodynamics without vasopressors.

## Introduction

Acute pancreatitis is a risk factor for intra-abdominal hypertension (IAH) and abdominal compartment syndrome (ACS) [1]. The incidence of IAH in severe acute pancreatitis is 60% and 30% for ACS [2-4]. The development of ACS in severe acute pancreatitis has a mortality rate of 50-75% [2-5]. Importantly, immediate management of IAH and early identification of ACS is critical for patient survival.

IAH is graded by the sustained levels of IAP typically measured by intra-bladder pressures. ACS is defined as a sustained intra-abdominal pressure (IAP) of >20 mmHg with new-onset organ dysfunction or failure [1]. The World Society of Abdominal Compartment Syndrome (WSACS) recommends IAP measurements every 4-6 hours in critically ill patients with one or more risk factors for the development of IAH/ACS [1]. Continuous IAP monitoring is feasible but is currently not a standard practice [6]. Many clinicians still rely on physical examination to assess IAP, though this has been proven to be highly inaccurate and clinically significant, IAH can be present in the absence of abdominal distension [7,8]. An assessment based on a perceived lack of abdominal tension can lead to a delay in ACS diagnosis and result in a poor prognosis. However, obtaining serial IAP measurements and accurate urinary output needed for timely diagnosis is challenging with conventional technology [9]. Manual IAP measurement requires appropriately setting up a transducer and entering the bladder catheter system to inject normal saline, obtaining the pressure through a hydrostatic column. Despite being technically possible, manual IAP monitoring is difficult to do accurately and time-consuming for an already burdened staff. Proper evaluation of urinary output is limited by the frequency of measurement, clearance of airlocks within the drainage tubing, and timely recording in the electronic medical record. The conventional urinary collection often results in urinary output being averaged over multiple hours based on collection frequency. The Accuryn Monitoring System (Potrero Medical, Hayward, CA) has been recently introduced; monitoring and measuring IAP, urinary output (UOP), and core body temperature [6,10] on a real-time basis.

ACS in a patient with severe alcohol-induced pancreatitis was detected early real-time trending of IAP and continuous monitoring of urinary output. The patient underwent decompressive laparotomy with hemodynamic stabilization and recovery of kidney function. An IRB approval was obtained by the Non-Research Data Use Committee (NRDUC) prior to publication.

## Case presentation

A 40-year-old male with a past medical history of chronic alcoholism, untreated hyperlipidemia, and hypertension presented to the emergency department with a one-day history of upper abdominal pain that progressively worsened and became unbearable to the patient. No associated vomiting or diarrhea, nor melena or hematemesis were reported by the patient. His blood pressure was 174/130 mmHg, and his oxygen saturation was 94% on room air. The patient acknowledged participating in a recent alcohol binge for the past three days.

**Figure 1:**
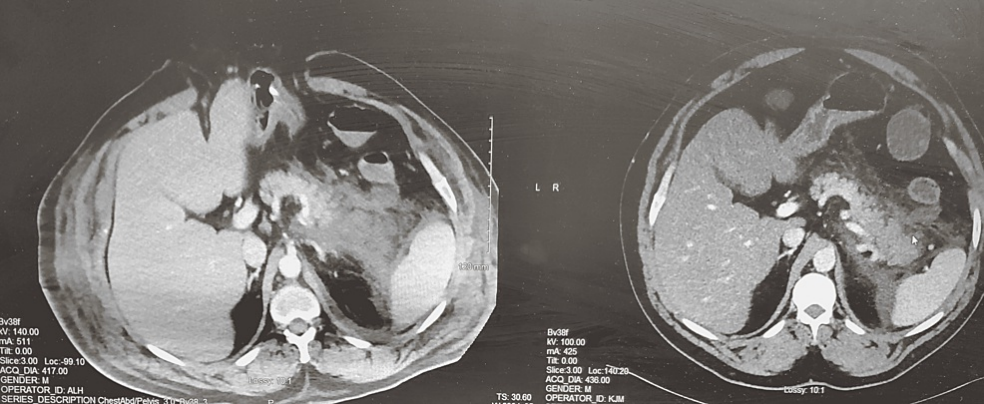
Computerized tomography of the abdomen showing a mildly enlarged distal portion of the pancreas with surrounding fluid and inflammation, consistent with pancreatitis.

A distended abdomen was visible upon examination and his epigastrium was tender upon palpitation. Computerized tomography (Figure 1) of the abdomen showed a mildly enlarged distal portion of the pancreas with surrounding fluid and inflammation, consistent with pancreatitis. The fluid and inflammatory changes extended from the body and tail of the pancreas into the left anterior pararenal space and adjacent to the spleen. The liver was enlarged with diffuse hepatic steatosis. The stomach, small bowel, large bowel, and appendix were normal. The gallbladder was normal, without any biliary dilation and the spleen was normal, too.

Pertinent laboratory values on admission are shown in Table 1.

**Table 1:** Pertinent laboratory values on admission. WBC: white blood count; ALT: alanine aminotransferase; AST: aspartate transferase

Laboratory Parameter	Value	Reference Range
Hemoglobin (g/dL)	20.5	13.5-17.5
Hematocrit (%)	59.3	38.3-48.6
WBC (counts/mcL)	16.2 × 10^3^	3.4-9.6 × 10^3^
Platelets (counts/mcL)	182 × 10^3^	135-317 × 10^3^
Total bilirubin (mg/dL)	3.9	0-1.2
ALT (IU/L)	346	7-55
AST (IU/L)	585	8-48
Lipase (units/L)	2694	24-151
Triglycerides (mg/dL)	1146	<150
Creatinine (mg/dL)	0.9	0.74-1.35

The patient was admitted to the hospitalist service for acute pancreatitis and was fluid resuscitated. Within 24 hours of admission, he was in distributive shock and became anuric. He progressed into respiratory and metabolic acidosis with worsening hypoxic respiratory failure. As a result, he was transferred to the ICU where he was intubated and catheterized with a smart sensing Foley (Accuryn Monitoring System, Potrero Medical, Hayward CA). His initial bladder pressure was 13 mmHg with paralysis. He was placed on high positive end-expiratory pressure (PEEP), low tidal volume ventilation strategy with neuromuscular blockade, and inhaled epoprostenol due to severe hypoxia with a PAO_2_/FiO_2_ ratio less than 100. Urinary output initially improved with volume resuscitation but then started to drop off by day three resulting in an 8.6 L positive fluid balance. At the same time as the decrease in urinary output, the bladder pressure was noted to have increased to over 20 mmHg, confirming ACS (defined as IAP>20 mmHg plus organ dysfunction). Surgical abdominal decompression was performed with immediate improvement in urinary output and further normalization of kidney function. Blood pressure and oxygenation stabilization allowed for weaning of vasopressors and PEEP. Temporary abdominal closure was achieved with open abdomen negative pressure therapy (ABTHERA™).

On hospital day three, the patient was treated with antibiotics for a high-grade fever with lung infiltrates and methicillin-sensitive *Staphylococcus aureus* in his tracheal aspirates. Although initially responsive, his fever recurred on day seven, and a CT of the abdomen revealed findings consistent with pancreatic necrosis. He was transferred to a tertiary center for further management of his pancreatitis on day eight of hospitalization. Chart review revealed that the patient was discharged home after recovery.

## Discussion

Acute pancreatitis is a major financial burden on the United States health system at a cost of 2.6 billion dollars/year [3]. It is the most common cause of inpatient hospitalization with a gastrointestinal diagnosis and its incidence is increasing [3]. For most patients, acute pancreatitis is a self-limited disease, but for the 15% of patients that develop severe disease, mortality rates of around 50% can occur, depending on the degree of organ failure [11]. When pancreatic necrosis with infection occurs, the mortality rate approaches 80% [12]. Severe acute pancreatitis is characterized by persistent organ failure (>48 hours) of the respiratory, cardiovascular, and renal systems that occurs due to a cytokine cascade-induced systemic inflammatory response [11]. Treatment is largely supportive, though a timely surgical intervention may be required if ACS progresses despite conservative management efforts.

A recent alcohol binge on top of already chronic alcoholism served as a trigger for his development of severe acute pancreatitis. Volume resuscitation for distributive shock and systemic inflammatory response syndrome (SIRS) led to respiratory failure and the development of IAH (13 mmHg) within the first 48 hours. Ongoing resuscitation over the next 24 hours contributed to the progression of ACS (IAPs over 20 mmHg), renal failure, and worsening respiratory status. Since this patient’s urine output and IAP could be monitored in real-time, ACS recognition was immediate, and surgical intervention took place without delay. The decompressive laparotomy led to a significant change in his clinical condition with a quick return of renal function and a significant improvement in oxygenation.

This dramatic clinical improvement speaks to the importance of early recognition of ACS and its necessity for timely surgical management. Optimal management and understanding of the abdominal compartment are a work in progress. The WSACS has made efforts to understand IAH/ACS and develop protocols and guidelines to optimize and standardize patient care. The WSACS defines IAH as a sustained or repeated pathological elevation in IAP > 12 mmHg which is graded by the level of IAP: grade I IAP 12-15 mmHg, grade II IAP 16-20 mmHg, grade III IAP 21-25 mmHg, and grade IV IAP > 25 mmHg. ACS is defined as a sustained IAP > 20 mmHg that is associated with new organ dysfunction or failure. The WSACS recommends serial or continuous IAP measurements when one or more risk factors for IAH/ACS are present [1]. While acute pancreatitis is a risk factor in itself for the development of IAH, pancreatitis-specific risk factors for the development of ACS have been described as a rise in creatinine, high Acute Physiology and Chronic Health Evaluation (APACHE)-II, or Glasgow Imrie scores, or as an elevation in the respiratory rate [1,13]. Management of IAH and ACS starts with medical management addressing a reduction of intraluminal contents, evacuation of intra-abdominal space-occupying lesions, improvements in abdominal wall compliance, and the optimization of fluid administration and systemic/regional perfusion. If the patient’s IAH/ACS is refractory to medical management and organ dysfunction or failure is present that can be attributed to elevated intra-abdominal pressures, surgical decompression as described in this case report is strongly encouraged.

Interestingly, the understanding and utilization of these WSACS guidelines remain low among critical care clinicians while an improvement in awareness of IAP, IAH, and ACS was noted in a recent survey [14]. More education and better tools are needed in this struggle to better understand the continuum of IAP-IAH-ACS and the importance of abdominal perfusion pressure to optimize patient outcomes.

In this case study, the Accuryn Monitoring System was utilized for urine output and IAP measurements. Frequent IAP monitoring with the Accuryn Monitoring System is possible due to the additional balloon sensor near the tip of the Foley, allowing for real-time measurements to appear on the bedside monitor. The Accuryn Monitoring System allows for continuous and accurate urine output measurements through an active drain line clearance mechanism. This mechanism prevents standing urine in the bladder or tubing. Having the ability to observe a) continuous and real-time urinary output and b) the kidneys’ response to certain interventions like fluids, vasopressors, or inotropes enables clinicians to get a real sense of the interactions of renal function, volume status, and cardiac output.

Severe acute pancreatitis continues to have a high mortality rate, although some progress has been made. This is the first case to our knowledge of acute pancreatitis successfully managed using real-time IAP and urinary output measurements. This case study demonstrates the value of continuously monitoring IAP and urinary output to quickly identify the development of ACS and AKI. This patient directly benefitted from timely intervention with the return of renal function and significantly improved hemodynamics. With early and frequent IAP monitoring capabilities, medical teams are able to recognize and address ACS immediately, allowing for prompt critical lifesaving measures to be undertaken for an improved patient prognosis. While commonly used guidelines by gastroenterological societies do not yet mention the benefit of routine IAP measurement [15,16], we follow other authors and the WSACS recommendations. Protocolized monitoring of IAP should routinely take place in high-risk patients such as those with severe acute pancreatitis, to allow for early detection and treatment of IAH and ACS [1,4,17].

## Conclusions

ACS contributes to the high mortality rate in severe acute pancreatitis. The clinical evaluation alone or in combination with intermittent only monitoring of IAP can contribute to delayed diagnosis of ACS. This case illustrates the importance of protocolized real-time monitoring of urinary output and IAP in severe acute pancreatitis allowing for a prompt diagnosis and treatment of ACS, ultimately leading to improved patient outcomes.
